# What drives the dynamics of employment growth in firms? Evidence from East Africa

**DOI:** 10.1186/s13731-023-00295-y

**Published:** 2023-05-24

**Authors:** Blessing Atwine, Ibrahim Mike Okumu, John Bosco Nnyanzi

**Affiliations:** 1 Research Analyst, Microeconomics Department, Economic Policy Research Centre (EPRC), Makerere University, Kampala, Uganda; 2 School of Economics, Makerere University, Kampala, Uganda; 3 Department of Economic Theory and Analysis, School of Economics, College of Business and Management Sciences, Makerere University, Pool Road, P. O. BOX 7062, Kampala, Uganda

**Keywords:** East Africa, Employment growth, Firm, Entrepreneur, Business environment, J2, L26, D2, O3, O5

## Abstract

This study examines the firm level drivers of employment growth in East Africa in which they are categorized as firm specific, entrepreneur specific and business environment characteristics. Using a cross-sectional World Bank Enterprise survey dataset and pooled Ordinary Least Squares estimation (OLS), the results indicate that; (1) employment growth is significantly associated with firm specific characteristics (employment growth is positively related to firm size and innovation while it is negatively associated with the age of the firm), (2) a weak business environment characterized by electricity outages, informal payments and poor court system undermines the firm’s ability to contribute to employment growth while strong business environment such as access to finance is positively associated with employment growth (3) employment growth is also positively influenced by managerial experience. Policy recommendations are suggested.

## Introduction

Unemployment is a concern to the developing economies because of its adverse effects on the social-economic welfare of the population by excluding them from accessing some social services as well as acting as a catalyst for income inequality as those employed continue earning and increasing their wealth stock while the unemployed become poorer (AfDB, 2018). According to Alrabba (2017), unemployment also raises the threat of social instability due to high crime rate and weaken private investment in human capital especially education because of the low discounted expected returns to such an investment (Mugisha, 2017). Besides, it is also said to hinder economic growth through blocking resource utilization Baah-Boateng (2013).

**Fig. 1:**
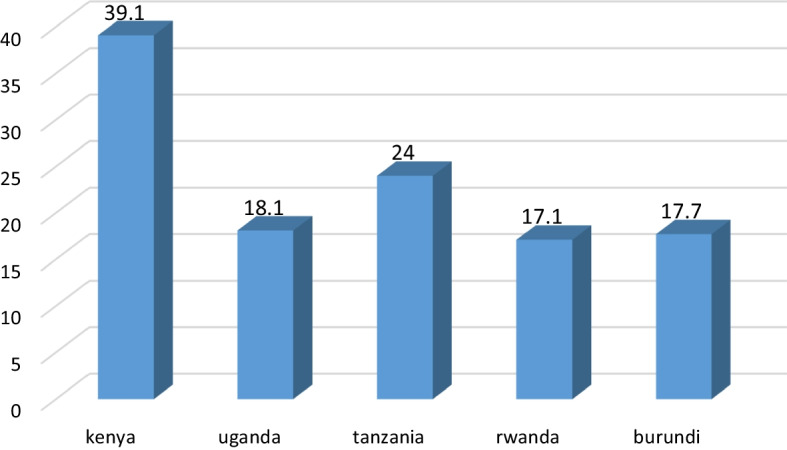
Unemployment rate among the East African countries in 2016. Source: African Development Bank (2018)

In the East African region, unemployment still remains one of the major pressing issues of concern for policy makers. During the period 2001–2018, the region registered tremendous growth and has indeed been labeled as the fastest growing region in the world. However, according to WDI (2019), during the same period that Uganda, Kenya and Tanzania collectively registered an impressive average annual growth rate in GDP of 5.9%, the overall growth in employment for the three East African countries lagged behind at an aggregate rate of 3.3%, with Uganda on top at 3.7%, followed by Kenya at 3.5% and Tanzania at 2.8%. For the entire East African countries, economic growth has stood at an average rate of 5.8% since 2010 (AfDB, 2019), while broad unemployment remained high. For example, for the five East African countries (Uganda, Kenya, Rwanda, Burundi, and Tanzania) this averaged at 36 per cent in 2016, with Kenya having the highest unemployment rate (39.1), followed by Tanzania with 24% Uganda with 18.1%, Burundi with 17.7% and Rwanda with 17.1% (see Fig. 1). Unemployment in the region is predicted to continue rising especially in the post-COVID-19 period, as many young graduates from the higher institutions of learning continue to join the labour market whose capacity to absorb the labour is already constrained. Thus, despite the high economic performance registered, a significant percentage of the population is not optimally benefiting from this growth as it is either underemployed or completely unemployed (East Africa Economic Outlook, 2018). What then drives the observed low levels of employment in the region?

The current study seeks to investigate the aforementioned question in greater detail. Certainly, the East African countries have endeavoured to address the problems of underemployment and unemployment, through designing strategies and programmes they deem fit to deal with the challenge at hand. Examples of such endeavours include but are not limited to the re-orientation of education systems to vocational and technical training, liberalization of labour markets, implementation of various special groups programmes such as youth livelihood programs as well as the Uganda Women Entrepreneurs Program (UWEP). These efforts notwithstanding, the issue of unemployment in the region is still worrisome. While the minimal impact of existing intermediations to curb low employment could be attributed to the high population growth rates (Uganda 3.26%, Kenya 2.48% Tanzania 3.08% Rwanda 2.35% and Burundi at 3.21%), other possible drivers of increasing unemployment pertain to labour market skills mismatch, high dependence on subsistence agriculture, as well as poor investment climate and the weak export promotion strategies, all of which limit the efforts of reducing unemployment (ILO, 2011). The issue then is how best countries in the region should manage the increasing labour force. Clearly, economists generally agree that employment growth is important for economic growth, though they differ on how this could be achieved. Investigating the determinants of employment growth is one way to provide guidance for evidence-based policies that aim at propelling employment to levels.

Existing empirical evidence on the drivers of employment growth has focused on a multitude of factors but produced mixed outcomes. Basically, three schools of thought can be detected in this literature: one focuses on firm-related factors (e.g. age, size, access to finance, ownership); the other on entrepreneur or founder-specific or owner-specific factors; and, the third argues for the environment-based factors (Schutjens & Wever, 2000). Nevertheless, the findings are divergent. For example, while some studies that focus on firm-specific factors have found a positive role of say, firm size, in employment growth (e.g. Yazdanfar & Salman, 2012; Okumu et al., 2019; Neumark et al., 2011; and, Ayyagari et al, 2014; Van Stel and Storey, 2004), others document a negative effect (e.g. Neumark et al., 2011; de Wit & de Kok, 2014). Yet, we equally observe that such factors do not have any definite relationship (e.g. Pyo, et al., 2016). Ambiguous findings are also not uncommon to come by. For example, while Adelino et al. (2017) argue that young firms create more employment, Hsieh and Klenow (2009) postulate that young firms are usually small and less productive thus create less employment. Whereas these studies are informative, most of them are based on developed and non-African. Only a few studies focus on African region, the main exceptions being Coniglio et al. (2015), Okumu et al. (2019) and, Naluwooza (2018), where focus is directed towards the role of innovations on employment growth. Nevertheless, studies that analyze the drivers of employment growth at a firm level from the East African perspective are scarce if not nonexistent. Additionally, it can be argued that in light of the above contradictory findings recorded in the empirical literature, the debate is inconclusive.

We contribute to literature in two ways. First, we analyze the firm level determinants of employment growth among firms in East Africa. Second, we examine the interaction effect of business environment characteristics including corruption, access to finance and electricity, as well as different firm size classes on employment growth. To achieve these objectives, the study uses the World Bank Enterprise Survey (WBES) data set on Uganda (2013), Kenya (2013) and Tanzania (2013), Rwanda (2011), and Burundi (2014) covering the 5 East African countries. Note that South Sudan is excluded from the sample for lack of data. The study covers 2754 manufacturing and services sector firms in EAC countries. As we later observe in the data section, in the survey, Tanzania had the largest number of firms surveyed (813 firms), followed by Kenya (781), Uganda (762) and Rwanda (241), and, Burundi (157). What this means is that the study is based on a well-representative sample, which to our knowledge no previous study has employed.

An analysis of the drivers of employment growth in East Africa is expectedly timely and beneficial to policy makers as well as to all interested parties in the labour market. Understanding the labour dynamics for the region would go a long way in guiding governments concerned on the important factors that can be focused on as they aim at achieving the United Nations Sustainable Development Goal 8, which is dedicated to the promotion of sustained, inclusive and sustainable economic growth, full and productive employment as well as decent work for all women and men, including young people and persons with disabilities (SDG 8.5).

In summary, the results reveal the following: (1) employment growth is significantly associated with firm specific characteristics, both positively (for the case of firm size and innovation) and negatively (in case of the age of the firm); (2) a weak business environment characterized by electricity outages, informal payments and poor court system undermines the firm’s ability to contribute to employment growth while strong business environment such as access to finance is positively associated with employment growth; (3) employment growth is also positively influenced by managerial experience.

The rest of the study is organized as follows. Section “Review of empirical literature” presents the theoretical and empirical literature related to the study. Section “Empirical strategy” presents the methodology adopted for the study including the conceptual framework, econometric model specification, data source, variable description and estimation procedure. Section “Results” presents the empirical findings of the study and their discussion. Section “Conclusions” comprises of summary of the study conclusions and recommendations.

## Review of empirical literature

As alluded to earlier, a number of studies have been carried out to examine the drivers of employment growth, albeit with mixed evidence. A plethora of these focus on firm-level determinants, viz., firm age and size, but also others such as innovation (both product and process innovation) and firm access to finance. It is however not clear the extent to which these influence employment growths among the East African countries, as there hasn’t been such a study conducted to-date, according to our knowledge. Nevertheless, related studies are here analyzed.

At the international level, Haltiwanger et al. (2013), using longitudinal data base of firms in the US, shows the importance of firm age in accounting for the relationship between firm size and employment growth. Their findings stipulate that business startups contribute substantially to both gross and net employment creation. However once firm age is included as a moderating variable, the evidence of a systematic relationship between firm size and employment growth breaks down. A later study by de Wit and de Kok (2014) however uses the dynamic classification method to analyze job creation with in different size classes of the 27 member states of the European Union. Interestingly, it is then found that small firms created more jobs relative to large firms, such that employment growth rate decreased with an increase in size. In confirmation of the latter finding, Hijzen et al. (2010) used firm-level data from 1997 to 2008 for almost all sectors in UK including services. Specifically, the authors show that small firms in the UK during the period under study accounted for a disproportionately large fraction of job creation and destruction relative to their share of employment.

Similarly, when Dogan et al. (2017) examined the relationship between firm size and net job creation using an extensive data set covering all non-farm Turkish businesses with 20 or more employees from 2003 to 2010, it was revealed that small firms (firms with employees between 20 and 100 employees) have higher mean job flow rates (job creation, job destruction and net job creation rates) than large firms. A similar conclusion was reached by Adelino et al. (2017), who, using an identification strategy that links shocks to local income to job creation in the nontradable sector and data from the US census quarterly workforce indicators derived from the longitudinal employer-household dynamics, reveals that new firms account for the bulk of net employment creation in response to local investment opportunities.

In contrast, Kuhn et al. (2016), used Danish employer-employee data from 2002 to 2007 that identify the start-ups and that cover almost the entire private sector to show that although start-ups are responsible for the entire overall net job creation, incumbents account for more than one-third of net job creation within high-skilled jobs. On the other hand, for the case of Great Britain as whole, however, Van Stel and Storey (2004) find no significant relationship between start-ups and employment creation in the 1980s. Yet, for the 1990s, a significant positive relationship for Great Britain as a whole is found, but for Scotland, which focused policy on start-ups, a negative relationship is found. Similarly, Voulgaris et al. (2005) for Greece find an inverse relationship between job creation and firm size. However, it is also found that older firms are the major job creators, while large firms are the major job destructors.

In another related study, Ayyagari, et al. (2014) used the World Bank Enterprise survey data on developing countries to conclude that small firms have the largest shares of job creation, and highest sales growth and employment growth, even after controlling for firm age. The latter findings did not differ much from a later study by Sintos (2018), who used the World Bank Enterprise survey data for 112 developing countries to show that small firms have the largest share of job creation compared to other firm size groups. Even after controlling for firm age, the author finds that small firms have the highest employment growth.

Similarly, at a country level, Acquisti and Lehmann (2000), studied job gross flows in Russia using large enterprise-level data sets from 1997 administrative records of firms in manufacturing and mining, construction and distribution and trade in four representative regions. Their results show that while small firms were the most successful at creating jobs, medium and large firms were mainly destroying them. In their analysis, the authors found that privatized firms fared no better than state-owned ones whilst new private firms’ outperformed firms with other ownership type as far as job creation is concerned. On the contrary, Kerr, Wittenberg and Arrow (2013) used the Quarterly Employment Survey on South Africa to document findings in support of larger firms in South Africa exhibiting higher employment growth rates compared to other size groups.

In other studies, Bigsten and Gebreeyesus (2007) for Ethiopia and Ayyagari et al. (2014) for a sample of 104 developing countries, job creation and employment growth was found negatively related to size and age, whereas labor productivity is observed affected positively by firm size and negatively by age, indicating that both size and age matter in the promotion of employment growth. Similarly, using a nonparametric regression approach**,** Banerjee and Jesenko (2016), reports that an inverse univariate relationship between firm size and net employment growth turns positive after controlling for firm age. Authors also note that young firms exhibit higher job creation and higher net employment growth rates than mature firms.

Existing literature additionally points to the role of access to finance, as an important factor of job creation. For example, Fowowe (2017) using data on Nigeria finds out that access to finance is important for business expansion, growth as well as job creation. On the other hand, a recent study by Okumu et al. (2019), used a cross-sectional World Bank Enterprise Survey dataset in which innovation is categorized as product innovation and process innovation to estimate the association between innovation and employment growth among manufacturing firms in Africa. Employing pooled ordinary least squares (OLS) estimation their results indicate that employment growth is positively and significantly associated with both process and product innovation although this study focuses only on the manufacturing sector. A similar finding is noticeable in a study by Naluwooza (2018).

The latter study is, to our knowledge, the only one that focuses on East Africa that we also consider in the current undertaking, albeit with significant differences, particularly with application of interactions and updated dataset. Moreover, in consideration of the previous literature, we examine a multitude of factors beyond innovations and the traditional ones capturing age and firm size. In addition, and to comprehensively examine the issue at hand, our model includes firm managerial experience, gender of top managers, and firm access to credit, corruption, competition, access to basic infrastructure (electricity) which we believe play an important role in influencing employment growth in the region, but also which have not been given attention in the existing literature, perhaps due to the fact that the panel data on firms that we employ and which captures these variables was not yet available.

## Empirical strategy

### Theoretical framework

As a basic theoretical framework, our study relies on the Law of Proportionate Effect (LPE) by Gibrat (1931), which states that the growth of firms is proportional to their size, and growth occurs at the same growth rate regardless of their initial size. In essence, the law posits that firms grow randomly every year, implying that growth of firms is independent of the size thus small or large firms have equal growth chances. In a formal framework, O’Farrell and Hitchens (1988) interpret Gibrat’s law in a stochastic model of firm growth constituting three elements.

The first is a constant growth rate of the market which is common to all firms. Let ${E}_{t}$ be firm employment size at time t and α be the constant growth rate of a firm, then

1 $$\frac{{E_{t + 1} - E_{t} }}{{E_{t} }} = \alpha$$

The second element is a systematic tendency for the employment growth of a firm to be related to its initial size $S_{t}$

2 $$\frac{{E_{t + 1} - E_{t} }}{{E_{t} }} = \alpha S_{t}^{\beta - 1}$$

$\beta = 1$ size has no effect on firm employment growth; $\beta \ne 1$ Size has an effect on firm employment growth.

The third element is a random growth term $\mu_{t}$ which enters the equation multiplicatively

3 $$\frac{{E_{t + 1} - E_{t} }}{{E_{t} }} = \alpha S_{t}^{\beta - 1} \mu_{t}$$

or

4 $$\Delta E_{it} = \alpha + \beta S_{i(t - 1)} + \mu_{i(t)}$$

The literature, both theoretical and empirical, indicates that there exist several factors that influence firm employment growth. These can be summarized as business environment, the firm specific and entrepreneur specific factors. Incorporating these and other variables in Eq. (4), we obtain Eq. (5):

5 $$\Delta E_{it} = \alpha \sum {X_{i} } + \beta S_{i(t - 1)} + \mu_{i(t)}$$

where $\sum {X_{i} }$ is a vector of other variables that both empirical and theoretical literature finds to influence firm employment growth.

### Empirical models

Equation (5) can then be interpreted to mean that firm employment growth is dependent on the size of the firm and other determinants, viz., business environment factors, other firm level factors and the entrepreneur specific factors.

In an extended form, we can now specify a model incorporating these general factors as in Eq. (6):

6 $$eg_{ijc} = \beta_{0} + \beta_{1} S_{i(t - 1)} + \beta_{2} K_{ijc} + \beta_{3} \varphi_{ijc} + \beta_{4} B_{ijc} + \mu_{ijc}$$

where $eg_{ijc}$ is the firm employment growth of firm $i$ in sector $j$ and country $c$;$S_{i(t - 1)}$ is firm size; $K_{ijc}$ is a vector of other firm specific characteristics,$\varphi_{ijc}$ captures the entrepreneur specific characteristics and $B_{ijc}$ is a vector of business environment characteristics $\mu_{ijc}$ is the error term and $\beta_{i}$ represents the coefficients (for $i$ ranging from 0 to n) while $i,c$ and $j$ capture the firm, sector and country respectively.

The baseline model for estimation is as specified below

7 $$\begin{gathered} eg_{ijc} = \beta_{0} + \beta_{1} age_{ijc} + \beta_{2} size_{ijc} + \beta_{3} innov_{ijc} + \beta_{4} ownership_{ijc} + \beta_{5} websiteuse_{ijc} + \\ \beta_{6} acc_{ijc} + \beta_{7} corr_{ijc} + \beta_{8} comp_{ijc} + \beta_{9} electricity_{ijc} + \beta_{10} courts_{ijc} + \beta_{11} gender_{ijc} + \\ \beta_{12} manager\exp_{ijc} + \beta_{13} formaltrain_{ijc} + \mu_{ijc} \\ \end{gathered}$$

where $acc$ is access to finance, $corr$ is corruption, $comp$ is competition, $innov$ is innovation, $gender$ represents gender of top manager,$manager\exp$ is managerial experience and $formaltrain$ is formal training.

We interact firm size and the different business environment aspects (corruption, access to finance and electricity outages) in which firms operate. This is to understand how the environment affects the different firm size classes and how this influences employment growth.

8 $$\begin{gathered} eg_{ijc} = \beta_{0} + \beta_{1} age_{ijc} + \beta_{2} size_{ijc} + \beta_{3} innov_{ijc} + \beta_{4} ownership_{ijc} + \beta_{5} websiteuse_{ijc} + \\ \beta_{6} acc_{ijc} + \beta_{7} corr_{ijc} + \beta_{8} comp_{ijc} + \beta_{9} electricity_{ijc} + \beta_{10} courts_{ijc} + \beta_{11} gender_{ijc} + \\ \beta_{12} manager\exp_{ijc} + \beta_{13} formaltrain_{ijc} + \beta_{14} \left( {size_{ijc} *corr_{ijc} } \right) + \mu_{ijc} \\ \end{gathered}$$

9 $$\begin{gathered} g_{ijc} = \beta_{0} + \beta_{1} age_{ijc} + \beta_{2} size_{ijc} + \beta_{3} innov_{ijc} + \beta_{4} ownership_{ijc} + \beta_{5} websiteuse_{ijc} + \\ \beta_{6} acc_{ijc} + \beta_{7} corr_{ijc} + \beta_{8} comp_{ijc} + \beta_{9} electricity_{ijc} + \beta_{10} courts_{ijc} + \beta_{11} gender_{ijc} + \\ \beta_{12} manager\exp_{ijc} + \beta_{13} formaltrain_{ijc} + \beta_{14} \left( {size_{ijc} *acc_{ijc} } \right) + \mu_{ijc} \\ \end{gathered}$$

10 $$\begin{gathered} g_{ijc} = \beta_{0} + \beta_{1} age_{ijc} + \beta_{2} size_{ijc} + \beta_{3} innov_{ijc} + \beta_{4} ownership_{ijc} + \beta_{5} websiteuse_{ijc} + \\ \beta_{6} acc_{ijc} + \beta_{7} corr_{ijc} + \beta_{8} comp_{ijc} + \beta_{9} electricity_{ijc} + \beta_{10} courts_{ijc} + \beta_{11} gender_{ijc} + \\ \beta_{12} manager\exp_{ijc} + \beta_{13} formaltrain_{ijc} + \beta_{14} \left( {size_{ijc} *electricity_{ijc} } \right) + \mu_{ijc} \\ \end{gathered}$$

### Definition and measurement of variables

The choice of variables considered in this study is guided by existing literature as earlier discussed and availability of data. The practical definitions are presented in Appendix: Table 7.

#### Firm-specific factors

In our model, firm size is measured by the number of employees of the firm. From the reviewed literature, it is accepted that small and medium sized firms are the main engine of employment growth (Li and Rama, 2015). We would therefore expect a positive relationship between firm size and employment growth.

Firm age is here defined as the difference between the time the survey was conducted and when the firm started operations. As in Adelino et al. (2017), firms less than 15 years are considered young. We hypothesize that on average young firms create less employment compared to their counterparts that have been in existence for longer periods. This argument is consistent with the learning model due to Jovanovic (1982) that younger firms learn over time which helps them to improve their performance. On the other hand, older firms can easily obtain resources that enable them to achieve higher performance (Audretsch, 1995).

Ownership structure which explains whether the firm is owned by locals or foreigners is another variable included in our model. Foreign owned firms are likely to be associated with lower employment growth in East Africa. This is because these firms tend to come in with their own labour and employ locals only for casual or informal jobs. This however has not been empirically tested. Locally owned firms are expected to employ more locals thus contributing more to employment growth compared to foreign owned firms.

Innovation is captured by process innovation and product innovation. The new products or processes introduced by a firm may involve a change in the production technique and a mix in the factor inputs which could either imply a reduction or an increase in the labor requirement. Specifically, product innovation which relates more to the demand-creation of a firm’s new product is expected to positively impact on employment growth (Capasso et al., 2015). On the other hand, process innovation may negatively impact on employment growth especially if it is labor saving in nature. This is because process innovation often entails a reduction in the unit cost of the factors of production, including the labor requirement necessary in achieving a unit output of a firm.

#### Business environment factors

It is here hypothesized that different firm size reacts differently to the business environments in which they operate (Aterido et al., 2010). Corruption is a business environment characteristic. Empirical literature shows that corruption impedes the growth potential of firms and their ability to create employment. This is because corruption results in paying exorbitant bribes which increases the cost of doing business (Yoong, 2017). Access to finance has been found to have a positive relationship with employment growth. This is because access to finance is important for business startups, expansion, growth as well as employment growth (Fowowe, 2017). Electricity is another business environment variable included in our model. Empirical evidence from past studies shows that unreliable electricity or constant power outages constrain the firm’s ability to grow and create employment (Aterido et al., 2011). Tax rate is also considered as a business environment factor in the model. A higher tax rate increases the cost of doing business in a country thus limiting firm entry and firm growth which in turn also limits employment growth. The expected sign is negative.

#### Entrepreneur-specific factors

Besides business environment factors, we include entrepreneur specific characteristics. These include formal training managerial experience and gender of top management. Formal training of a firms’ employees improves their capabilities and efficiency, this results into an improvement in the firm’s productivity and profitability leading to expansion and employment growth (Naluwooza, 2018). On the other hand, managerial experience, is expected to be positively related to employment growth as the more the years of experience, the better will be the skills of management that leads to firm growth and hence employment growth.

### Data source and description

To explain the firm level determinants of employment growth in the East African region, the research uses data from the World Bank Enterprise survey (WBES) for five East African community member states; Uganda, Kenya, Tanzania, Rwanda and Burundi. The WBES dataset is a global firm survey that undertakes face-to-face interviews with top managers and business owners in 139 countries that are engaged in non-agricultural formal sector. The manufacturing and service sectors are the primary business sectors of interest and formal registered firms with 5 and more employees are targeted for interview. The survey uses the two stage stratified random sampling strategy in which all population units are grouped within homogeneous groups and simple random samples are selected within each group. This method allows computing estimates for each of the strata with a specified level of precision while population estimates can also be estimated by properly weighting individual observations. The data employed covers a period of 2013 for Uganda, Tanzania and Kenya; 2011 for Rwanda and 2014 for Burundi. The WBES dataset follows the global standardized methodology. The reason for the difference in years for the countries considered is due to the fact that data collection was carried out at varying time intervals in the respective countries. Data for the countries considered is pooled to form one dataset that is used in the analysis. The WBES data covers information on various aspects of business environment and investment climate of economies with topics ranging from innovation, sales and supplies performance, finance, infrastructure and business-government relations. The country dummy variables are included in the study to control for fixed effects. Because our surveys were conducted in different years for different countries these dummies also control for years of sampling across space and time (see Taylor and Naude, 2000).

Table 1 reports the description of our data. As per the table, the average employment growth rate in East Africa is at 3.46% with a minimum of − 46.8% and a maximum of 5%. Employment growth is highest in Tanzania at 6.55% and lowest in Kenya at 1.09%. Employment growth rate in Rwanda, Burundi and Uganda is at 4.39%, 3.32% and, 2.33% respectively. For firm specific characteristics, with regards to age, firms are categorized as young, mature and older. Young firms are those that have been in existence for less than 5 years, mature firms are between 6 and 15 years while older firms are those that have existed for at least 16 years. The average firm age is at 16.87 years with the oldest at 107 years and youngest at 1 year. Ugandan, Tanzanian and Burundian firms are on average of the same age at 15 years while Kenyan firms are the oldest at 22 years with the youngest from Rwanda at 11 years. For size, 46% of the firms are small, Tanzania has the highest proportion of small firms at 33% followed by Uganda, Kenya, Rwanda and Burundi, at 31%, 23%, 7%, and 5% respectively. The large firms are only 20% with Kenya having the highest share at 45% and Burundi the lowest at only 3%. Regarding firm Ownership, majority of the firms in EAC countries are domestically owned at 88% while 12% of the firms were owned by foreigners. Tanzania had the highest share of domestically owned firms 31% followed by Uganda and Kenya at 28% whereas Rwanda and Burundi account for 8% and 5% respectively. With regards to export status 23% of the sampled firms were involved in exporting their products and services. Kenya has the largest proportion of exporting firms at 46% followed Uganda at 25.3%, Tanzania at 20%, Burundi at 4.3% and the least share from Rwanda at 4.1%. Regarding location 61% of the firms in the EAC region are located in the official capital city of the country (Kampala for Uganda, Nairobi for Kenya, Kigali for Rwanda, Bujumbura for Burundi and Dodoma for Tanzania). Uganda had the highest percentage with 35% of the firms located in Kampala, followed by Kenya which had 32% of the firms in Nairobi, then Rwanda had 22% of the firms in Kigali and Burundi had 10% of the firms in Bujumbura.

**Table 1:** Descriptive statistics. Source: Own computation based on WBSE 2019

Variable	Observations	Mean	sd	min	max
Employment growth	2754	0.0346	0.127	− 0.468	0.500
Firm age	2754	16.87	13.69	1	107
Size(small = base category)					
Medium	2754	0.308	0.462	0	1
Large	2754	0.129	0.335	0	1
Ownership (domestic = category)					
Foreign ownership	2622	0.125	0.330	0	1
Export status (1 = yes)	2754	0.230	0.421	0	1
Managerial experience	2624	14.87	9.476	1	57
Location (2 = outside official city)	2754	0.611	0.488	0	1
Formal training (1 = yes)	2721	0.366	0.482	0	1
Access to finance (2 = no)	2565	1.695	0.460	1	2
Business licensing (1 = yes)	2677	0.681	0.466	0	1
Tax rate (1 = obstacle)	2754	0.660	0.474	0	1
Gender (1 = female)	2749	0.140	0.347	0	1
Informal payments	1664	2.091	8.813	0	100
Power outage	2754	6.922	2.421	4	15
Corruption (1 = obstacle)	2642	0.753	0.431	0	1
Competition (1 = yes)	2579	0.676	0.468	0	1
Country (1 = others)	2754	0.723	0.447	0	1
Sector (1 = service)	2754	0.583	0.493	0	1

With regards to entrepreneurship characteristics, only 36.6% of firms in the region had offered formal training to their permanent. Kenya had the majority at 34%, Uganda 24%, Tanzania 23%, Rwanda 14% and Burundi 5% of firms reporting to have offered formal training to employees. Regarding to managerial experience, on average managers from the region have 15 years of experience with the most experienced managers coming from Kenya at 18.36 years, Uganda Rwanda, Burundi and Tanzania managers on average have 13 years of experience.

Furthermore, the overall proportion of firms that had access to finance was 31%. Burundi had the smallest share at 4%, followed by Rwanda at 7% then Tanzania at 21% with Uganda at 29% and Kenya had the highest number of firms with access to finance at 38%. In addition, the most experienced firm manager in the region had 57 years of experience and one year for the least experienced manager. The average experience of the firm manager was found to be 14.87 years. The most experienced managers were from Kenya with an average of 18 years. Uganda, Rwanda and Tanzania had relatively the same experience at 13 years. The least experience was from Burundi at 12 years.

Regarding the Business Environment, 66% of firms reported tax rate to be an obstacle in their operations. Most of these firms were from Kenya at 33%, Uganda at 30% and Tanzania at 25%. In Rwanda, 10% of the firms reported tax rate as an obstacle while in Burundi this accounted for only 2%. Considering corruption, 75.3% of firms in the EAC region are reported to have faced corruption in dealing with public officials. Uganda had the highest proportion of firms to have encountered corrupt public officials at 33.2% followed by Kenya at 29.2% and Tanzania at 28.9%. Burundi and Rwanda, reported the lowest incident of corruption at 4.4% and 4.1% respectively. In addition, 67.6% of firms in the EAC region are reported to have faced competition from informal firms, 36% of these firms were from Uganda, 28% from Tanzania, 25% from Kenya while 7% and 5% of the firms were from Rwanda and Burundi respectively. Regarding business licensing 68% 0f the firms in the region reported to have faced an obstacle in buying business licenses. Tanzania faced the highest obstacle at 33.8% followed by Uganda at 32%, Kenya at 26%, Burundi at 4.38% and Rwanda faced the lowest obstacle at 3.6%.

The sector dummy is a control variable that capture sector specific factors. From the table above it follows that 58.3% of the firms in the study are in the service sector relative to 41.7% that are in the industrial sector.

Appendix: Table 5 shows pairwise correlation of explanatory variables. As evidence in the table, all the correlation coefficients are below the 0.8 standard measure, which could imply an absence of multicollinearity between variables.

### Estimation strategy

This study employs the pooled Ordinary Least Square estimation technique (OLS) to estimate the above Eqs. 7, 8, 9 and 10. The choice of this technique is because of its simplicity, convenience and the fact that it has been successfully used by other studies and gives out meaningful results (Stock and Watson, 2003; Wooldridge, 2015). The key assumption is that the error term *u* has zero mean given any value of the independent variable *x* (Wooldridge, 2015). However, because of the cross-sectional nature of the data used in the study which brings in the possibility of endogeneity, the OLS technique would be produce biased estimates. One source of fear of endogeneity is the possibility that firms experiencing growth are more likely to engage in innovation activities (Fu et al., 2017). Likewise, innovation may also result in employment growth thus causing a challenge of reverse causality. Therefore, we carried out an endogeneity test for the innovation variables using the Durbin-Wu-Hausman test. The results confirmed no existence of endogeneity problem, leading to the adoption of the ordinary OLS method.

## Results

Regarding firm age, the results reveal that firm age and employment growth are inversely related as indicated by the negative and statistically significant coefficients for firm age (see Table 2, Models 1). In other words, younger firms experience significantly faster growth in employment as compared to older firms. This finding implies that young firms are critical for higher employment creation rates than older firms and this is in support of earlier findings of Ayyagari et al. (2014), Kuhn et al. (2016) and Adelino et al. (2017) who also argue that young firms employ a large share of workers and create more employment opportunities than mature firms.

**Table 2:** Regression results for the firm level determinants of employment growth. Source: Own calculation

Variables	Model (1)	Model (2)	Model (3)	Model (4)
Firm specific factors				
Firm age	− 0.001*** (0.000)	− 0.000* (0.000)	− 0.001*** (0.000)	− 0.001*** (0.000)
ln age squared	− 0.029** (0.013)	− 0.029** (0.013)	− 0.022* (0.012)	− 0.028** (0.012)
Size (small = base category)				
Medium	0.026*** (0.008)			
Large	0.056*** (0.011)			
Ownership (domestic = base category)				
Foreign Ownership	0.005 (0.010)	0.009 (0.010)	0.006 (0.010)	0.005 (0.009)
Use website (1 = yes)	0.003 (0.007)	0.012* (0.007)	0.002 (0.007)	0.002 (0.007)
Innovation (1 = yes)	0.009** (0.0034)	0.010 (0.007)	0.008 (0.007)	0.009 (0.007)
Business environment factors				
Access to finance (2 = No)	0.020*** (0.007)	0.014* (0.007)		0.020*** (0.007)
Courts (1 = yes)	− 0.012* (0.007)	− 0.012* (0.007)	− 0.013* (0.007)	− 0.012 (0.007)
Competition (1 = yes)	0.008 (0.007)	0.005 (0.007)	0.007 (0.007)	0.008 (0.007)
Electricity (1 = yes)	− 0.015* (0.008)	− 0.016* (0.008)	− 0.016* (0.009)	
Informal payments	− 0.001** (0.000)		− 0.001*** (0.000)	− 0.001** (0.000)
Business licensing (1 = yes)	0.004 (0.007)	0.003 (0.007)	0.004 (0.008)	0.004 (0.008)
Entrepreneur specific factors				
Gender (1 = female)	− 0.017* (0.009)	− 0.020** (0.009)	− 0.017* (0.009)	− 0.017** (0.009)
Formal training (1 = yes)	− 0.010 (0.007)	− 0.006 (0.007)	− 0.012* (0.007)	− 0.010 (0.007)
Managerial experience	0.014** (0.006)	0.007 (0.008)	0.004 (0.008)	0.005 (0.008)
Interactions				
Small X informal payments		− 0.002*** (0.001)		
Medium X informal payments		− 0.001 (0.001)		
Large X informal payments		0.002 (0.003)		
Small X access to finance			0.000 (0.000)	
Medium X access to finance			0.021** (0.010)	
Large X access to finance			0.044*** (0.012)	
Small X electricity obstacle				− 0.005 (0.011)
Medium X electricity obstacle				0.018 (0.013)
Large X electricity obstacle				− 0.044*** (0.015)
Constant	0.153*** (0.058)	0.170*** (0.059)	0.148*** (0.057)	0.141** (0.056)
Country fixed effect	Yes	Yes	Yes	Yes
Sector fixed effect	Yes	Yes	Yes	Yes
Observations	1300	1300	1300	1300
R^2^	0.068	0.049	0.066	0.070

Robust standard errors in parentheses; ***p < 0.01, **p < 0.05, *p < 0.1

For firm size, on the other hand, medium and large firms are found to positively and significantly influence employment growth compared to small firms. This finding implies that as we move from small to medium and to large firms, employment growth increases by 0.028 folds and 0.066 folds respectively (Table 2, Model 1). The finding is consistent with earlier study by Kerr, Wittenberg and Arrow (2013) who focuses on South Africa. Note that after disaggregating by sector we find that in both service and manufacturing firms, medium and large firms positively and significantly influence employment growth compared to the small firms (see Tables 3 and 4).

**Table 3:** Firm level determinants of employment growth in the service sector. Source: own calculation

Variables	Model (1)	Model (2)	Model (3)	Model (4)
Firm specific factors				
Firm age	− 0.000 (0.000)	− 0.001*** (0.000)	− 0.001** (0.000)	− 0.001*** (0.000)
ln age squared	− 0.027 (0.021)	− 0.027* (0.016)	− 0.028* (0.016)	
Size (small = base category)				
Medium	0.021* (0.012)			
Large	0.036** (0.015)			
Ownership (domestic = base category)				
Foreign ownership	0.018 (0.014)	− 0.010 (0.014)	− 0.005 (0.014)	− 0.007 (0.014)
Use website (1 = yes)	0.006 (0.011)	0.001 (0.010)	− 0.004 (0.010)	− 0.000 (0.010)
Innovation (1 = yes)	0.018 (0.011)	0.001 (0.009)		0.001 (0.009)
Business environment factors				
Courts (1 = yes)	− 0.011 (0.012)	− 0.013 (0.009)	− 0.014 (0.009)	− 0.015* (0.009)
Electricity (1 = yes)	− 0.007 (0.015)	− 0.019* (0.010)	− 0.019* (0.010)	
Informal payments	− 0.000 (0.001)		− 0.002*** (0.001)	− 0.002*** (0.001)
Access to finance (2 = no)	0.015 (0.011)	0.024** (0.010)		0.023** (0.010)
Business licensing (1 = yes)	− 0.002 (0.012)	0.009 (0.009)	0.009 (0.009)	0.007 (0.009)
Competition (1 = yes)	0.017* (0.011)	0.001 (0.009)	0.001 (0.009)	− 0.000 (0.009)
Entrepreneur specific factors				
Managerial experience	− 0.005 (0.013)	0.010 (0.009)	0.008 (0.009)	0.011 (0.009)
Gender (1 = female)	− 0.011 (0.015)	− 0.020* (0.011)	− 0.022** (0.011)	− 0.019* (0.011)
Formal training (1 = yes)	− 0.020* (0.011)	− 0.001 (0.009)	0.001 (0.009)	0.001 (0.009)
Interactions				
Small X informal payments		− 0.003*** (0.001)		
Medium X informal payments		− 0.001 (0.001)		
Large X informal payments		0.000 (0.005)		
Small X access to finance			0.008 (0.012)	
Medium X access to finance			0.044*** (0.015)	
Large X access to finance			0.125*** (0.024)	
Small X electricity obstacle				(0.000) 0.034*
Medium X electricity obstacle				(0.032) − 0.008
Large X electricity obstacle				(0.014) 0.050**
Constant	0.138 (0.099)	0.155** (0.072)	0.169** (0.072)	0.007 (0.021)
Country specific effect	Yes	Yes	Yes	Yes
Observations	559	741	758	741
R^2^	0.054	0.103	0.104	0.112

Standard errors in parentheses; *** p < 0.01, ** p < 0.05, * p < 0.1

**Table 4:** Firm level determinants of employment growth in the manufacturing sector. Source: Authors calculations

Variables	Model (1)	Model (2)	Model (3)	Model (4)
Firm specific factors				
Firm age	− 0.000 (0.000)	− 0.000 (0.000)	− 0.000 (0.000)	− 0.001 (0.000)
ln age squared	− 0.027 (0.021)	− 0.028 (0.021)	− 0.025 (0.021)	− 0.028 (0.021)
Size (small = base category)				
Medium	0.021* (0.012)			
Large	0.036** (0.015)			
Ownership (domestic = base category)				
Foreign ownership	0.018 (0.014)	0.022 (0.014)	0.018 (0.014)	0.020 (0.014)
Use website (1 = yes)	0.006 (0.011)	0.013 (0.011)	0.005 (0.011)	0.007 (0.011)
Innovation (1 = yes)	0.018 (0.011)	0.019* (0.011)	0.019* (0.011)	0.017 (0.011)
Business environment factors				
Access to finance (2 = No)	0.015 (0.011)	0.010 (0.011)		0.015 (0.011)
Courts (1 = yes)	− 0.011 (0.012)	− 0.011 (0.012)	− 0.011 (0.012)	− 0.012 (0.012)
Competition (1 = yes)	0.017* (0.011)	0.015 (0.011)	0.017 (0.011)	0.019* (0.011)
Electricity (1 = yes)	− 0.007 (0.015)	− 0.007 (0.015)	− 0.008 (0.015)	
Informal payments	− 0.000 (0.001)		− 0.000 (0.001)	− 0.000 (0.001)
Business licensing (1 = yes)	− 0.002 (0.012)	− 0.003 (0.012)	− 0.003 (0.012)	− 0.001 (0.012)
Entrepreneur specific factors				
Gender (1 = female)	− 0.011 (0.015)	− 0.014 (0.015)	− 0.011 (0.015)	− 0.012 (0.015)
Formal training (1 = yes)	− 0.020* (0.011)	− 0.017 (0.011)	− 0.020* (0.011)	− 0.020* (0.011)
Managerial experience	− 0.005 (0.013)	− 0.001 (0.013)	− 0.006 (0.013)	− 0.006 (0.013)
Interactions				
Small X informal payments		− 0.000 (0.001)		
Medium X informal payments		− 0.001 (0.001)		
Large X informal payments		0.001 (0.004)		
Small X access to finance			0.019 (0.019)	
Medium X access to finance			0.031 (0.020)	
Large X access to finance			0.067*** (0.023)	
Small X electricity obstacle				0.049* (0.028)
Medium X electricity obstacle				− 0.037** (0.015)
Large X electricity obstacle				0.000 (0.000)
Constant	0.138 (0.099)	0.154 (0.099)	0.129 (0.100)	0.133 (0.100)
Country fixed effects	Yes	Yes	Yes	Yes
Observations	559	559	559	559
R^2^	0.054	0.043	0.058	0.057

Standard errors in parentheses; ***p < 0.01, **p < 0.05, *p < 0.1

Similarly, and as expected, employment growth is positively and significantly associated with innovation. Specifically, firms that engage in innovation activities have the capacity to increase employment growth by 0.008 folds compared to firms that do not engage in innovation (see Model 1 in Table 2). Earlier studies by Okumu et al. (2019), Bogliacino (2014), Cirera and Sabetti (2016) and Fukao et al. (2017) inter alia, document similar findings. However, once we disaggregate by sector, the results reveal differential outcomes. Specifically, while innovation has a neutral effect on employment in the service sector (see Table 3), employment growth is significantly and positively influenced by innovation in the manufacturing sector (see Table 4, Model 2).

With regard to the business environment, the results presented in Table 2, model 2, reveal that employment growth is undermined among firms that experience electricity outages compared to those that do not. Since electricity outages represent the quality of public infrastructure, and is an integral input of production, the adverse effects on employment growth would imply that shortages electricity supply exert adverse impact on firm’s productivity and profits. Since firms employ labour until at least the marginal product of labor is equal to wages, a falling marginal product of labour resulting from electricity outages will have a negative impact on labour demand thereby reducing employment growth. An earlier study by Aterido et al. (2011) reports a similar finding. Interacting firm size and electricity outages, results reveal that small and large firm’s contribution to employment growth is negatively influenced by electricity obstacles (see Table 2, Model 2), however in the service sector electricity outages in the small and large firms has a neutral influence on employment growth. However medium firms facing electricity obstacles negatively influence employment growth in the manufacturing sector (see Table 4, Model 2).

Further evidence in Table 2 also reveals that firms that face obstacles in dealing with courts of law limit their employment creation capacity by 0.012 folds compared to firms facing no such obstacle (Model 1 in Table 2). Since the court systems proxy the quality of institutional framework, this result suggests that a weak institutional framework undermines employment growth. This finding is in agreement with the findings of Surinach et al. (2007) who found out that poor institutions limit employment growth. However, at a sectoral level, a poor institutional framework is found to significantly and negatively influence employment growth whereas this relationship is neutral in the manufacturing sector (see Tables 3 and 4).

With regards to corruption, firms that make a lot of informal payments are observed to perform poorly (by 0.001 folds) relative to those that do not (see Model 1 in Table 2). Perhaps, this is because corruption hampers all economic activities through increased transaction costs, greater uncertainty and less transparency in markets. Additionally, it can be argued that corruption could erode the profits and resources of the firm through bribery demands, that would in turn hinder firm’s expansion and thus employment growth. These results are consistent with the findings of other studies such as Yoong (2017). Interestingly, the interaction of firm size and corruption reveals that while corruption is neutral to large firms in both sectors, it negatively and significantly hurts small firm’s capacity to contribute to employment growth in the service sectors while it’s neutral to medium firms (see Tables 2, 3 and 4, Model 2).

Turning to gender, employment growth is found to be negatively and significantly influenced by female top management. Specifically, firms that are managed by females perform poorly in terms of employment growth by 0.017 folds compared to firms that are managed by males (see Model 1 in Table 2). Regardless of the sectoral differences, female top managers negatively contribute to the firm’s capacity to create employment. Perhaps these findings would reflect the fact that there are too few females in top management positions to impact on employment in East Africa. It is not news that gender-based employment segregation is rooted in social norms and deepened by discrimination and educational segregation.

Pertaining to managers’ experience, we established that an extra year of experience obtained by a firm manager leads to an increase in employment growth by 0.014 folds (see Model 1, Table 2). This implies that as managers acquire experience, their capabilities and efficiency increase which in turn increases the firm’s productivity. This in turn will increase the demand for labor as in input thus leading to an increase in employment growth. These results are consistent with the findings of Jansen et al. (2002) who also finds a positive significant relationship between employment growth and managerial experience.

On the other hand, firms that undertake formal training of their full time employees appear to reduce employment growth by 0.012 folds compared to those that do not undertake formal training (see Model 3, Table 2). This negative relationship could be from the fact that employees who receive formal training become more efficient and can take on multiple roles, thus reducing the possibility of hiring new labour force.

Regarding access to finance, the results confirm our earlier hypothesis that firms which can easily access finance increase employment growth compared to those that do not have access to finance (see Model 1, Table 2). Specifically, the increase is by about by 0.020 times. By implication, access to finance is not only important for business expansion and growth but also employment creation. These findings are in agreement with earlier findings in Aterido and Hallward-Driemeir (2010) and Fowowe (2017) inter alia. One important note is that the interactions between size and access to finance show that small firms with no access to finance contribute less to employment growth compared to large firms. The lack of access to finance for small firms’ means that they are unable to expand and grow thus cannot hire more labour units while medium and large firms are neutral to obstacles regarding access to finance (see Table 2, 3 and 4, Model 3).

## Conclusions

The major aim of the current study was to examine the firm level determinants of employment growth in East Africa. Using the World Bank Enterprise Survey data, the results based on pooled OLS reveal that employment growth is significantly and positively associated with some firm specific characteristics such as size and both process and product innovation while it has a negative significant relationship with firm age. While process and product innovation are found to complement each other in influencing employment growth, they individually have no impact on employment growth. The findings also indicate that a weak business environment characterized by power outages and corruption in form of informal payments inversely affects employment growth. However, findings also reveal that a good business environment such as access to finance improves the firm’s ability to create more jobs thus increasing employment growth. Results indicate that employment growth is positively associated with entrepreneur factors such as managerial experience. That is to say that managers that are experienced have the ability to expand their business and create more jobs. On the other hand, female top manager create less employment and formal training of employees enables them to multitask thus reducing the firms need to hire more labour. Thus formal training and gender show a negative significant association with employment growth.

We suggest firm managers and owners to vigorously embrace innovation just as policy makers should ensure that innovation supporting policies are in place to further employment growth. Government policies that focus on R&D investments and those targeting the development and strengthening of linkages between different research institutions and firms ought to be fully supported. Also, the study revelation that for firms to grow, they must overcome credit constraints, calls for policies aimed at overcoming obstacles in obtaining finance as well as making financial services accessible and affordable through viable credit mechanisms to support and strengthen the capacity of firms to create employment. Similarly, the findings reflect the urgent need for the East African countries to provide a conducive business environment in terms of quality and quantity of public infrastructure, particularly reliable energy, as well as anti-corruption measures and legal institutions, to enable firms expand, grow and create more jobs. Particularly important for the services as well as manufacturing sectors, stakeholders need to acknowledge the pivotal role of access to finance in resolving the unemployment issue in East Africa. There is an urgent need for creditors to have access to information on a regional basis for example. The regional integration of credit information would call for the development and implementation of EAC protocols and standards but also joint ventures with trustworthy credit agencies from other countries. In practical terms, the removal of all credit constraints through well-coordinated policies would optimize employment growth in the services as well as the manufacturing sectors. We further advocate for policies to improve access, affordability and availability of electricity, alongside the removal of all forms of corruption including but not limited to informal payments as doing so would facilitate employment growth in both the services and manufacturing sectors. The government provision of funding and a conducive environment to boost innovative ventures to spur competition can go a long way in driving employment growth for the manufacturing sector in the region. Finally, if unemployment in East Africa is to be resolved, the study findings point to the macroeconomic policies that target firm size as a key factor in employment growth of the services and manufacturing sectors. Policies that reduce the average size of establishments would detrimentally impact on employment growth in these sectors, as large sized-firms as well as medium ones are found to significantly reduce unemployment in each of the two sectors.

Nevertheless, the cross section nature of our dataset prevents us from controlling for other time variant variables that may affect employment growth but are not captured in the survey. Perhaps, as more rounds of the World Bank Enterprise Survey (WBES) are conducted, future studies may employ panel techniques to control for this unobserved heterogeneity. We further suggest that future studies could specifically look at firms that drive employment growth in East Africa, as well as the impact of labour market institutions and skill shortages on firm dynamism. These areas were outside the scope of our analysis despite their close linkages to the current study.

## Data Availability

World Bank Enterprise survey dataset used for the study is available online.

## Appendix

(see Tables 5, 6, 7).

**Table 5:** Correlation matrix. Source: Own calculation

	Variables	1	2	3	4	5	6	7	8	9	10	11	12	13	14	15	16	17	18
1	Employment growth	1																	
2	Firm age	− 00.02	1																
3	size (2 = medium)	0.04*	0.06*	1															
4	size (3 = large)	0.13*	0.26*	− 0.25*	1														
5	Gender (1 = female)	− 0.05*	− 0.05*	− 0.06*	− 0.06*	1													
6	Ownership (2 = foreign)	0.05*	0.04*	0.07*	0.16*	− 0.02	1												
7	Innovation	0.02	0.09*	0.09*	0.12*	− 0.01	0.04*	1											
8	Export status (1 = yes)	0.01	0.22*	0.07*	0.30*	− 0.03	0.17*	0.13*	1										
9	Managerial experience	0.01	0.36*	0.05*	0.14*	− 0.10*	0.01	0.07*	0.08*	1									
10	location (2 = outside city)	0.01	0.03*	− 0.06*	− 0.10*	− 0.03	− 0.14*	− 0.10*	− 0.00	− 0.02	1								
11	Formal training (1 = yes)	− 0.00	0.10*	0.06*	0.20*	− 0.00	0.09*	0.30*	0.15*	0.05*	− 0.10*	1							
12	Access to finance (2 = no)	0.02	− 0.13*	− 0.10*	− 0.16*	− 0.00	− 0.03	− 0.12*	− 0.14*	− 0.08*	0.12*	− 0.14*	1						
13	Tax rate (1 = obstacle)	− 0.01	0.01	0.00	− 0.01	0.02	− 0.03	− 0.01	0.03	− 0.01	− 0.05*	0.00	− 0.00	1					
14	Informal payments	− 0.06*	0.00	0.03	− 0.06*	− 0.02	− 0.01	− 0.01	0.06*	− 0.01	0.09*	− 0.02	− 0.02	0.01	1				
15	Power outage	0.06*	− 0.09*	− 0.00	− 0.10*	0.01	0.00	− 0.11*	− 0.09*	− 0.12*	0.12*	− 0.11*	− 0.01	− 0.25*	− 0.00	1			
16	Competition (1 = yes)	− 0.00	− 0.05*	− 0.05*	− 0.10*	0.01	− 0.06*	0.08*	− 0.09*	0.05*	− 0.06*	0.01	0.04*	− 0.03	0.00	0.03*	1		
17	Country (0 = Uganda)	0.05*	0.10*	0.04*	0.07*	− 0.04*	− 0.03	− 0.07*	0.02	0.04*	0.13*	0.06*	− 0.12*	− 0.05*	− 0.15*	− 0.02	− 0.26*	1	
18	Sector (1 = service)	− 0.02	− 0.21*	− 0.06*	− 0.18*	0.03*	− 0.07*	− 0.05*	− 0.22*	− 0.13*	− 0.00	− 0.04*	0.11*	0.06*	0.00	0.02	0.07*	0.003	1

**p* < 0.05

**Table 6:** Descriptive statistics by country. Source: Author calculations based on WBES survey data

Country	Variables	N	Mean	sd	Min	Max
Uganda	Employment growth	762	0.0233	0.114	− 0.468	0.500
	Firm age	762	14.60	10.29	1	86
	Size(small = base category)					
	Medium	762	0.274	0.446	0	1
	Large	762	0.0866	0.281	0	1
	Ownership (domestic = category)					
	Foreign ownership	741	0.143	0.350	0	1
	Export status (1 = yes)	762	0.210	0.408	0	1
	Manager experience	727	13.76	8.433	1	41
	location (2 = out city)	762	0.503	0.500	0	1
	Formal training (1 = yes)	755	0.317	0.465	0	1
	Access to finance (2 = no)	684	1.792	0.406	1	2
	Tax rate (1 = yes)	762	0.706	0.456	0	1
	Business licensing (1 = yes)	760	0.771	0.420	0	1
	Gender (1 = female)	762	0.167	0.373	0	1
	Informal payments	374	4.559	12.18	0	80
	Power outages	762	7	0	7	7
	competition (1 = yes)	709	0.879	0.327	0	1
	country (1 = others)	762	0	0	0	0
	sector (1 = service)	762	0.580	0.494	0	1
Burundi	Employment growth	157	0.0332	0.0974	− 0.310	0.433
	Firm age	157	15.08	13.73	1	87
	Size(small = base category)					
	Medium	157	0.408	0.493	0	1
	Large	157	0.0764	0.267	0	1
	Ownership (domestic = category)					
	Foreign ownership	156	0.199	0.400	0	1
	Export status (1 = yes)	157	0.172	0.379	0	1
	Manager experience	156	12.78	9.458	1	46
	Location (2 = out city)	157	0.293	0.457	0	1
	Formal training (1 = yes)	157	0.312	0.465	0	1
	Access to finance (2 = no)	156	1.378	0.487	1	2
	Tax rate (1 = yes)	157	0.274	0.447	0	1
	Business licensing (1 = yes)	149	0.537	0.500	0	1
	Gender (1 = female)	157	0.172	0.379	0	1
	Informal payments	119	0.992	3.590	0	20
	Power outages	157	15	0	15	15
	Competition (1 = yes)	141	0.582	0.495	0	1
	Country (1 = others)	157	1	0	1	1
	Sector (1 = service)	157	0.624	0.486	0	1
	Employment growth					
Kenya	Employment growth	781	0.0109	0.0980	− 0.432	0.500
	Firm age	781	22.76	17.78	1	107
	Size(small = base category)					
	Medium	781	0.342	0.475	0	1
	Large	781	0.204	0.403	0	1
	Ownership (domestic = category)					
	Foreign ownership	715	0.113	0.317	0	1
	Export status (1 = yes)	781	0.374	0.484	0	1
	Manager experience	761	18.36	10.86	1	57
	Location (2 = out city)	781	0.553	0.497	0	1
	Formal training (1 = yes)	775	0.434	0.496	0	1
	Access to finance (2 = no)	742	1.609	0.488	1	2
	Tax rate (1 = yes)	781	0.777	0.416	0	1
	Business licensing (1 = yes)	777	0.614	0.487	0	1
	Gender (1 = female)	781	0.102	0.303	0	1
	Informal payments	530	1.885	7.619	0	100
	Power outages	781	5	0	5	5
	Competition (1 = yes)	749	0.573	0.495	0	1
	Country (1 = others)	781	1	0	1	1
	Sector (1 = service)	781	0.519	0.500	0	1
Rwanda	Employment growth	241	0.0439	0.114	− 0.289	0.485
	Firm age	241	11.23	9.881	1	52
	Size(small = base category)					
	Medium	241	0.373	0.485	0	1
	Large	241	0.154	0.361	0	1
	Ownership (domestic = category)					
	Foreign ownership	239	0.213	0.411	0	1
	Export status (1 = yes)	241	0.108	0.311	0	1
	Manager experience	235	13.40	8.897	1	42
	Location (2 = out city)	241	0.0373	0.190	0	1
	Formal training (1 = yes)	241	0.577	0.495	0	1
	Access to finance (2 = no)	231	1.511	0.501	1	2
	Tax rate (1 = yes)	241	0.743	0.438	0	1
	Business licensing (1 = yes)	237	0.274	0.447	0	1
	Gender (1 = female)	241	0.199	0.400	0	1
	Informal payments	192	0.172	0.990	0	10
	Power outages	241	4	0	4	4
	Competition (1 = yes)	220	0.541	0.499	0	1
	Country (1 = others)	241	1	0	1	1
	Sector (1 = service)	241	0.689	0.464	0	1
Tanzania	Employment growth	813	0.0655	0.161	− 0.400	0.500
	Firm age	813	15.35	10.78	1	96
	Size(small = base category)					
	Medium	813	0.269	0.444	0	1
	Large	813	0.0984	0.298	0	1
	Ownership (domestic = category)					
	Foreign ownership	771	0.0752	0.264	0	1
	Export status (1 = yes)	813	0.157	0.364	0	1
	Manager experience	745	13.29	8.086	1	50
	Location (2 = out city)	813	1	0	1	1
	Formal training (1 = yes)	793	0.295	0.456	0	1
	Access to finance (2 = no)	752	1.814	0.390	1	2
	Tax rate (1 = yes)	813	0.555	0.497	0	1
	Business licensing (1 = yes)	754	0.817	0.387	0	1
	gender (1 = female)	808	0.127	0.334	0	1
	Informal payments	449	1.390	9.212	0	100
	Power outages	813	8	0	8	8
	Competition (1 = yes)	760	0.645	0.479	0	1
	Country (1 = others)	813	1	0	1	1
	Sector (1 = service)	813	0.609	0.488	0	1
Total	Employment growth	2754	0.0346	0.127	− 0.468	0.500
	Firm age	2754	16.87	13.69	1	107
	Size (small = base category)					
	Medium	2754	0.308	0.462	0	1
	Large	2754	0.129	0.335	0	1
	Ownership (domestic = category)					
	Foreign ownership	2622	0.125	0.330	0	1
	Export status (1 = yes)	2754	0.230	0.421	0	1
	Manager experience	2624	14.87	9.476	1	57
	Location (2 = out city)	2754	0.611	0.488	0	1
	Formal training (1 = yes)	2721	0.366	0.482	0	1
	Access to finance (2 = no)	2565	1.695	0.460	1	2
	Tax rate (1 = yes)	2754	0.660	0.474	0	1
	Business licensing (1 = yes)	2677	0.681	0.466	0	1
	Gender (1 = female)	2749	0.140	0.347	0	1
	Informal payments	1664	2.091	8.813	0	100
	Power outages	2754	6.922	2.421	4	15
	Competition (1 = yes)	2579	0.676	0.468	0	1
	Country (1 = others)	2754	0.723	0.447	0	1
	Sector (1 = service)	2754	0.583	0.493	0	1

**Table 7:** Variables and measurement

Variable name	Unit	Description
Dependent variable		
Employment growth	Continuous	Is measured as the difference between permanent employees in the last fiscal year and 3 years before the survey divided by the firm’s average permanent employees during the same period $EG_{ijc} = {{\left( {EG_{ijct} - EG_{ijc(t - 3)} } \right)} \mathord{\left/ {\vphantom {{\left( {EG_{ijct} - EG_{ijc(t - 3)} } \right)} {\left( {{{EG_{ijc(t - 3)} } \mathord{\left/ {\vphantom {{EG_{ijc(t - 3)} } 2}} \right. \kern-0pt} 2}} \right)}}} \right. \kern-0pt} {\left( {{{EG_{ijc(t - 3)} } \mathord{\left/ {\vphantom {{EG_{ijc(t - 3)} } 2}} \right. \kern-0pt} 2}} \right)}}$ Where $t$ and $t - 3$ represent last fiscal year and 3 years before the survey, respectively
Independent variables		
Firm specific characteristics		
Age	Continuous	This calculated as the difference between the year the survey was taken in each country and the year when the firm began its operations
Size	Category	This is generated from the number of full time permanent employees and is categorized into small (20 and below employees), medium (20–100 employees) and large (more than 100 employees)
Research and development	Dummy	Defined as to whether the firm spent on R&D in the previous fiscal year taking on the value of ‘1’ if the firm spent on R&D and ‘0’ if the firm did not spend on R&D
Ownership	Dummy	Measured as to whether the firm is owned by private foreign individual taking on a value of ‘2’ or domestically owned with a value of ‘1’
Innovation	Dummy	Refers to whether a firm introduced both a new product and process in the last fiscal year; taking on a value of ‘1’ if the firm introduced both a new product and process
Website use	Dummy	Takes on the value of ‘1’ if the firm has its own website or zero otherwise
Entrepreneur characteristics		
Managers experience	Continuous	Defined as the number of years of experience of the firm’s top manager
Gender of top management	Dummy	Takes on the value of ‘1’if the top manager was female and ‘0’ otherwise
Formal training	Dummy	Takes on the value of ‘1’ if the firm had formal training programs for its full time employees in the last fiscal years and ‘0’ otherwise
Business environment		
Electricity	Dummy	Measures whether firms experienced any power outage in the last fiscal year prior to the survey, taking on the value of ‘1’ is a firm experienced any obstacles and zero otherwise
Access to finance	Dummy	Defined in the dataset as to whether a firm found an obstacle in accessing finance taking on a value of ‘1’ if the firm found any obstacles and ‘2’ otherwise
Informal payments	Continuous	Defined as the percentage of total annual sales paid in informal payments to public official in the dataset
Competition	Dummy	Takes on two measure in the dataset; the first asking firms whether they face more than 5 competitors and the second asking firms whether they face any competition from informal or unregistered firms. In the study, competition takes on a value of ‘1’ if the firm faced competition and ‘0’ otherwise
Business licensing		This relates to how much business licensing is an obstacle to firms and takes on the value of ‘0’ for those without any obstacles and ‘1’ for those firms that found any obstacles
Court system		Defined in the dataset as to whether a firm found an obstacle in accessing courts of law taking on a value of ‘1’ if the firm found any obstacles and ‘0’ otherwise
Control variables		
Country	Dummy	Defined as the country dummy with Uganda taking on a value of ‘0’ while Burundi, Kenya, Rwanda and Tanzania are taking on a value of ‘1’ ‘2’ ‘3’ and ‘4’respectively
Sector	Dummy	Two sectors are considered in the study. That is; the manufacturing sector taking on the value of ‘1’ and service sector which takes on the value of ‘0’
